# Forecasting of Landslide Displacement Using a Probability-Scheme Combination Ensemble Prediction Technique

**DOI:** 10.3390/ijerph17134788

**Published:** 2020-07-03

**Authors:** Junwei Ma, Xiao Liu, Xiaoxu Niu, Yankun Wang, Tao Wen, Junrong Zhang, Zongxing Zou

**Affiliations:** 1 Three Gorges Research Center for Geo-Hazards of the Ministry of Education, China University of Geosciences, Wuhan 430074, China; majw@cug.edu.cn (J.M.); nxx@cug.edu.cn (X.N.); zouzongxing@cug.edu.cn (Z.Z.); 2 Faculty of Engineering, China University of Geosciences, Wuhan 430074, China; yankun_wang@cug.edu.cn (Y.W.); zjr@cug.edu.cn (J.Z.); 3 School of Geosciences, Yangtze University, Wuhan 430100, China; wentao200840@yangtzeu.edu.cn

**Keywords:** landslide displacement, predictive uncertainty, ensemble prediction, probability combination scheme, quantile regression neural networks (QRNNs), kernel density estimation (KDE)

## Abstract

Data-driven models have been extensively employed in landslide displacement prediction. However, predictive uncertainty, which consists of input uncertainty, parameter uncertainty, and model uncertainty, is usually disregarded in deterministic data-driven modeling, and point estimates are separately presented. In this study, a probability-scheme combination ensemble prediction that employs quantile regression neural networks and kernel density estimation (QRNNs-KDE) is proposed for robust and accurate prediction and uncertainty quantification of landslide displacement. In the ensemble model, QRNNs serve as base learning algorithms to generate multiple base learners. Final ensemble prediction is obtained by integration of all base learners through a probability combination scheme based on KDE. The Fanjiaping landslide in the Three Gorges Reservoir area (TGRA) was selected as a case study to explore the performance of the ensemble prediction. Based on long-term (2006–2018) and near real-time monitoring data, a comprehensive analysis of the deformation characteristics was conducted for fully understanding the triggering factors. The experimental results indicate that the QRNNs-KDE approach can perform predictions with perfect performance and outperform the traditional backpropagation (BP), radial basis function (RBF), extreme learning machine (ELM), support vector machine (SVM) methods, bootstrap-extreme learning machine-artificial neural network (bootstrap-ELM-ANN), and Copula-kernel-based support vector machine quantile regression (Copula-KSVMQR). The proposed QRNNs-KDE approach has significant potential in medium-term to long-term horizon forecasting and quantification of uncertainty.

## 1. Introduction

As one of the most common natural hazards in the world, landslides pose a significant threat to public health and safety. According to statistics, landslides have affected 4.8 million people and caused 18,275 deaths during the period of 2009–2019 [1]. Landslide displacement prediction, which provides the necessary information to determine the extent of ongoing hazard, has proven to be the most cost-saving risk reduction measure [2,3,4]. However, landslide displacement prediction is complex and remains a key challenge in natural hazard research. This challenge arises because landslides are nonlinear, dynamic systems, and the associated movements can be induced by different causes, such as geological factors [5], hydrological factors [6,7], morphological factors, and human activities [4,8].

A large number of efforts in the literature have focused on the precise prediction of landslide displacement [9]. Currently, approaches used for landslide displacement prediction are categorized as physical modelling approaches and data-driven approaches [10]. Physical models (also known as white-box models), which rely on detailed descriptions of landslide mechanism processes, can provide clear physical explanations of landslides. The commonly used physical models include the tertiary creep model [11], the Hayashi model [12], and the general creep model [13]. Those physical models require numerous expensive geotechnical characterizations of the materials involved in landslides and therefore may be applicable only in limited cases [14].

Data-driven models differ from physical models because a characterization of the actual landslide mechanism processes is not fully required. Thus, the data-driven models are also known as black-box models. The main advantage of data-driven models is that the trained models can be easily updated on the basis of new and more recent data.

Data-driven models include but are not limited to statistical methods, artificial neural networks (ANNs), support vector machines (SVMs) [15], and extreme learning machines (ELMs) [16]. Owing to their capacity to approximate arbitrary, nonlinear, and dynamic systems with high precision, data-driven models achieve good model performance in the prediction of landslide displacement.

Despite their widespread application, the output of most existing data-driven models is a single estimate for each prediction horizon. These single estimates, which provide deterministic values, are referred to as point predictions [3]. The defining characteristic of a point prediction is its accessibility with regard to understanding and operation. The main drawback of point prediction is that it only provides the prediction error, with no information regarding the associated predictive uncertainties, which limits the use of point prediction in decision-making applications.

The predictive uncertainties consisting primarily of input uncertainty, parameter uncertainty, and model uncertainty could be substantial. It is highly desirable to know the degree of uncertainty that is associated with a particular point prediction and convert the point prediction into informative resources for emergency landslide risk management [3,17]. Only limited studies have examined the quantification of uncertainty associated with landslide displacement prediction by constructing prediction intervals (PIs). The output of a PI is an interval composed of upper and lower bounds, where we expect the predictive value of the series to fall within some (prespecified) probability, which is deemed the PI nominal confidence (PINC). A hybrid approach based on an echo state network and mean-variance estimation was proposed by Yao et al. [18] to measure the uncertainty in landslide deformation prediction and perform interval prediction. A bootstrap-based approach was proposed by Ma et al. [4] to perform interval prediction of landslide displacement. Wang et al. [2] proposed a direct interval prediction using least squares support vector machines or the construction of PIs of landslide displacement. Kernel-based support vector machine quantile regression (KSVMQR) was utilized in [3] for quantification of the predictive uncertainty of landslide displacement.

However, the traditional methods have certain disadvantages in displacement prediction and quantification of predictive uncertainty. For example, the bootstrap-based approach requires significantly high computational costs, especially for large datasets [2]. Additionally, the performances of SVM-based approaches are sensitive to the choice of kernel type and parameter values [19]. Therefore, more efforts still need to be made for the improvement of prediction performance and quantification of the predictive uncertainty.

Ensemble prediction, a state-of-the-art artificial intelligence technique, aims to improve prediction robustness and accuracy and uncertainty quantification [20,21]. Ensemble prediction has been successfully applied in a variety of fields, including prediction performance improvement and uncertainty quantification of remaining useful life [22], bankruptcy [23], shear capacity of reinforced-concrete deep beams [24], residential electricity consumption [25], wind power [26], flood susceptibility [27,28], and landslide susceptibility [29].

In this study, a probability-scheme combination ensemble prediction that employs quantile regression neural networks and kernel density estimation (QRNNs-KDE) was proposed for robust and accurate prediction and uncertainty quantification of landslide displacement. The Fanjiaping landslide with long-term and near real-time monitoring data was selected as a case study to explore the performance of the QRNNs-KDE approach. The deformation characteristics were clarified for fully understanding the triggering factors.

## 2. Methodology

### 2.1. Description of Uncertainty Sources

Predictive uncertainty in data-driven models consists primarily of input uncertainty, parameter uncertainty, and model uncertainty [30,31,32].

The input uncertainty is related to the input data uncertainty and the input variable section uncertainty. The input data uncertainty is primarily due to measurement and sampling error and environmental noise. The input variable section uncertainty accounts for uncertainty inherent in the selection of input variables from the candidate data set. For physical models, the required inputs are pre-determined, being consistent with considered rheological models. However, for data-driven models, the selection of input variables is problem-dependent and cannot be determined in advance. Only major and relevant variables are selected as final inputs to train the data-driven model. The selection of the variables to include in a data-driven model from the original data set is inherently uncertain, especially when the input candidate pool is very large. For example, in data-driven models that utilize decomposition algorithms, only a portion of the decomposed sub-components are selected as input variables. The candidate input pool, which consists of sub-components, increases very quickly with the decomposition level and potentially increases the input variable selection uncertainty.

The parameter uncertainty refers to the uncertainty in the model parameter vector and mainly arises from the inability to identify a unique set of best parameters for the model [33].

Model uncertainty arises primarily from the model structure uncertainty and model error. Model structure uncertainty is associated with the specific model setting of learning algorithms, such as the polynomial order in polynomial regression models, the number of hidden nodes in an ANN or ELM, and the type of kernel function in an SVM. The input uncertainty may also account for model structure uncertainty, because different input variables “automatically" produce different model structures. Model error refers to the difference between two model estimates with respect to the corresponding target and is caused by the inability to reproduce the real processes.

### 2.2. Ensemble Prediction

**Figure 1:**
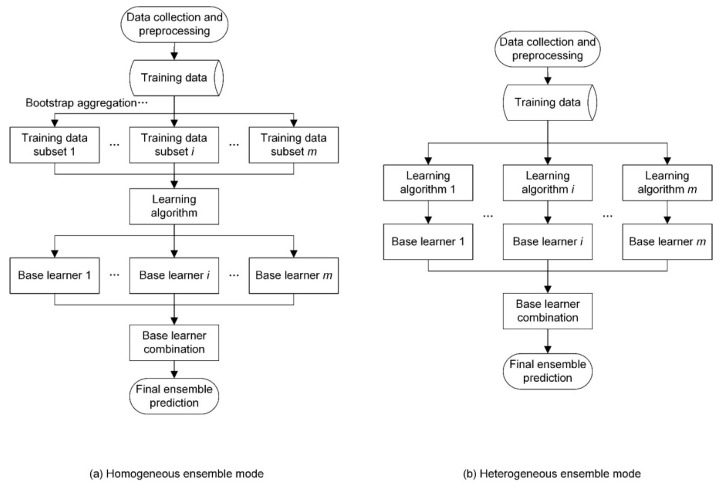
General framework for ensemble prediction models. (**a**) Homogeneous ensemble model and (**b**) heterogeneous ensemble model.

Ensemble prediction is not a specific learning algorithm but a strategic combination of multiple predictions into a single output with a model combination process [21]. Based on the selection of the learning algorithm, ensemble prediction models can be further classified into homogeneous and heterogeneous ensemble models (Figure 1). A homogeneous ensemble model generates multiple learners with the same learning algorithm on different training datasets, which are produced by manipulating the original training data (schematic illustrated in Figure 1a). Bootstrap aggregation, also known as bagging for short, is the most straightforward and widely used method of manipulating the training dataset. By contrast, a heterogeneous ensemble model generates multiple learners with different learning algorithms on the same training data set (schematic illustrated in Figure 1b).

The base learner combination is the main step in the ensemble prediction model. Summation and averaging are simple combination schemes. A more general approach involves assigning a weight to each base learner. In the present study, a heterogeneous ensemble model was built based on QRNNs and KDE. QRNNs serve as base learning algorithms to produce multiple base learners, and the probability combination scheme based on KDE is used to combine the base learners into the final ensemble prediction.

### 2.3. Quantile Regression Neural Network

#### 2.3.1. Quantile Regression

Quantile regression is a common statistical technique for conducting inferences concerning conditional quantile functions [34,35]. More formally, any real-valued random variable *Y* may be characterized by its distribution function as follows:

(1) F(y)=Prob(Y≤y)

whereas for any 0<τ<1,

(2) Q(τ)=inf{y:F(y)≥τ}

is called the τth quantile of *Y*.

Given a data set $\left(x_{i}(t),Y(t)\right)$ for i=1,2,⋯,I and t=1,2,⋯,N, the linear quantile regression can be expressed as follows:

(3) $${\hat{Y}}_{\tau }(t)=\sum_{i=1}^{I}\theta_{i}x_{i}(t)+b$$

where 0<τ<1 is the quantile, and *b* is an error with zero expectation.

The estimated parameters $\theta_{i}$ can be approximated by minimizing a sum of the asymmetrically weighted absolute residual cost functions, which are expressed as follows:

(4) $$E_{\tau }=\frac{1}{N}\sum_{t=1}^{N}\rho_{\tau }(Y(t)-{\hat{Y}}_{\tau }(t))$$

where Y(t) is the observation at time *t* and $\rho_{\tau }$ is the check function, which is also known as the pinball loss function and is defined as follows:

(5) $$\rho_{\tau }(x)=\{\begin{array}{ll} \tau x & \;\mathrm{if}\;x\geq 0\\ (\tau -1)x & \;\mathrm{if}\;x<0 \end{array}$$

#### 2.3.2. Quantile Regression Neural Network

Given inputs $x_{i}(t)$ and an output Y(t), the output from a QRNN is calculated as follows:

Consider a hidden-layer transfer function h(⋅); the output from the *j*-th hidden-layer node $g_{j}(t)$ is given by applying the hidden-layer transfer function to the inner product between $x_{i}(t)$ and hidden-layer weights $w_{ij}^{\left(h\right)}$ plus the hidden-layer bias $b_{j}{}^{\left(h\right)}$, which can be calculated as follows:

(6) $$g_{j}(t)=h(\sum_{i=1}^{I}x_{i}(t)w_{ij}^{\left(h\right)}+b_{j}^{\left(h\right)})$$

An estimate of the conditional τ-quantile ${\hat{y}}_{\tau }(t)$ is

(7) $${\hat{Y}}_{\tau }(t)=f(\sum_{j=1}^{J}g_{j}(t)w_{j}^{\left(o\right)}+b^{\left(o\right)})$$

where $w_{j}^{\left(o\right)}$ are the output-layer weights, $b^{\left(o\right)}$ is the output-layer bias, and f(⋅) is the output-layer transfer function. The transfer function h(⋅) and f(⋅) are usually set as the hyperbolic tangent sigmoidal and linear function, respectively [36].

As an alternative method to prevent overfitting, weight delay regularization for the magnitude of the input-hidden layer weight can be applied by setting a penalty with a nonzero value.

### 2.4. Kernel Density Estimation (KDE)

Nonparametric density estimation is the process of fitting a parametric density model of a random variable without making the assumption that the density belongs to a particular parametric family [37,38]. Various methods have been proposed for nonparametric density estimation, e.g., k-nearest neighbors method, Parzen windows, histogram, and KDE [38]. In the domain of nonparametric density estimation, the K-nearest neighbors method has a very limited scope of practical applications due to its very poor performance. The Parzen windows method presents slightly better performance but also produces discontinuities (stair-like curves) that are quite annoying in practice [38]. A histogram is a simple form of the nonparametric density estimation. However, it suffers serious and noticeable drawbacks. First, the resulting visualization strongly depends on the choice of binning. Second, the natural feature of the histogram is discontinuity, which causes extreme difficulty if derivatives of the estimates are required.

Fortunately, those abovementioned drawbacks can be easily eliminated by using KDE [38,39]. In fact, KDE has been extensively studied and has become the most popular method in nonparametric density estimation. Given a random sample $Y_{1},Y_{2},\cdots ,Y_{m}$, the value of the density at the point y estimated by the KDE method is given by the following:

(8) $$\hat{f}(y,h)=\frac{1}{mh}\sum_{i=1}^{m}K(\frac{y-Y_{i}}{h})$$

where h is the bandwidth with positive real value and K(⋅) is the kernel function. In this study, the most effective Epanechnikov kernel [38] was adopted and expressed as

(9) $$K(y)=\frac{3}{4}(1-y^{2})\mathbb{R}(\left|y\right|\leq 1)$$

where ℝ(⋅) is the indicator function, that is, ℝ(y∈A)=1 for y∈A and ℝ(y∈A)=0 for y∉A.

The selection of bandwidth parameter is a crucial issue in KDE. The bandwidth parameter influences the smoothness of the KDE curve and also determines the tradeoff between the bias and variance. In general, the smaller the bandwidth, the smaller the bias, and the larger the variance. A number of methods have been proposed to find the optimal bandwidth, such as Silverman’s rule of thumb and the Sheather-Jones method. Silverman’s rule of thumb bandwidth with a Gaussian kernel and Epanechnikov kernel can be computed as follows:

(10) $$h^{optimal}\approx 1.06\hat{\sigma }n^{-\frac{1}{5}}$$

(11) $$h^{optimal}\approx 2.34\hat{\sigma }n^{-\frac{1}{5}}$$

where $\hat{\sigma }$ is the estimation of σ (standard deviation of the input data) [38].

### 2.5. Ensemble Prediction Employing QRNNs and KDE

**Figure 2:**
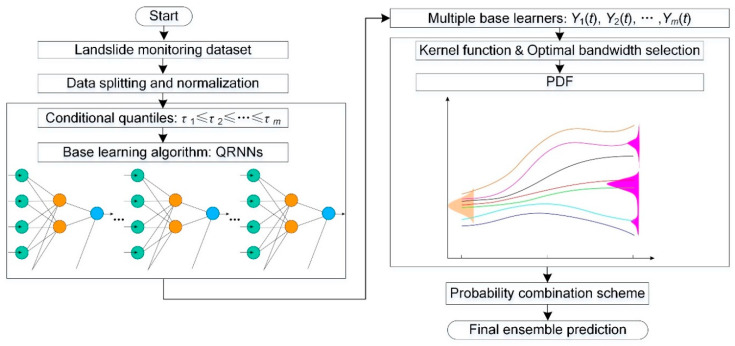
The overall flowchart of ensemble prediction based on the quantile regression neural networks and kernel density estimation (QRNNs-KDE) approach.

The proposed ensemble prediction employing QRNNs and KDE is shown in Figure 2. The QRNNs-KDE approach consists of four stages: (1) data splitting and normalization, (2) QRNN modelling, (3) probability density function (PDF) estimation by KDE, and (4) final ensemble prediction.

Data splitting and normalization: The original landslide monitoring dataset is divided into training data and testing data. The training data are used for model construction, and the testing data are used to evaluate the performance of the constructed model. To eliminate the influence of dimensional data, the training data and testing data are first normalized in the range of 0 to 1.

QRNNs modelling: QRNNs serve as base learning algorithms to generate multiple base learners $Y_{1}(t),Y_{2}(t),\cdots ,Y_{m}(t)$ by applying a finite number of conditional quantities $\tau_{1}\leq \tau_{2}\leq \cdots \leq \tau_{m}$ within the domain 0<τ<1, e.g., τ = 0.01, 0.02, …, 0.98, 0.99. The base learners of landslide displacement are obtained after renormalizing the outputs from the QRNNs approach. To avoiding overfitting in QRNNs modelling, a penalty parameter with nonzero value is applied.

PDF estimation by KDE: Multiple base learners from the QRNNs base model are treated as the input for KDE to estimate the probability density function (PDF) of the base learners. The kernel function and bandwidth influence the shape of the KDE curve. An appropriate kernel function and an optimal bandwidth should be chosen to best match the features of the original dataset.

Final ensemble prediction: In the present study, the final ensemble prediction was obtained through a probability combination scheme as follows:

(12) $$u_{t}=\sum_{i=1}^{m}p_{i}(t)Y_{i}(t)$$

where $p_{i}(t)$ is the probability value of the *i*-th base learner and $Y_{i}(t)$ is obtained from the KDE for monitoring period t.

### 2.6. Evaluation Metrics and Uncertainty Quantification

In this study, five indices—coefficient of determination (*R*^2^) MSE, RMSE, NRMSE, and MAPE—were applied to assess the performance of point prediction. R^2^, MSE, RMSE, NRMSE, and MAPE are defined as

(13) $$R^{2}={\left[\frac{\sum_{t=1}^{N}\left(u_{t}-\bar{u})({\hat{u}}_{t}-\bar{\hat{u}}\right)}{\sqrt{\sum_{t=1}^{N}{\left(u_{t}-\bar{u}\right)}^{2}{\left({\hat{u}}_{t}-\bar{\hat{u}}\right)}^{2}}}\right]}^{2}$$

(14) $$MSE=\frac{\sum_{t=1}^{N}{\left({\hat{u}}_{t}-u_{t}\right)}^{2}}{N}$$

(15) $$RMSE=\sqrt{\frac{\sum_{t=1}^{N}{\left({\hat{u}}_{t}-u_{t}\right)}^{2}}{N}}$$

(16) $$NRMSE=\sqrt{\frac{\sum_{t=1}^{N}{\left({\hat{u}}_{t}-u_{t}\right)}^{2}}{\sum_{t=1}^{N}u_{t}{}^{2}}}$$

(17) $$MAPE=\frac{1}{N}(\sum_{t=1}^{N}\left|\frac{{\hat{u}}_{t}-u_{t}}{u_{t}}\right|)\times 100\%$$

where ${\hat{u}}_{t}$ and $u_{t}$ denote the *t*-th predictive value and observation, respectively, and $\bar{u}$ and $\bar{\hat{u}}$ denote the mean of the observation and the mean of the predictive value, respectively.

In the present study, the associated predictive uncertainties were quantified with PIs. After the above procedures, full PDFs of the future landslide displacement were achieved. An interval prediction with a (1−α)×100% confidence interval can be obtained from the α/2 and 1−α/2 quantiles of the obtained PDF. The α level, also called the significance level, ranges from 0 to 1 and is the probability of not capturing the value of the parameter. The predictive values of the α/2 quantity and 1−α/2 quantity are set as the upper bound ($U_{t}^{1-\alpha }$) and lower bound ($L_{t}^{1-\alpha }$), respectively. For example, a 90% central PI can be obtained from the 0.05 and 0.95 quantiles of the PDF. The upper bound and lower bound of the 90% confidence level correspond to the predictive values of the 0.95 and 0.05 quantiles of the obtained PDF.

The prediction interval coverage probability (PICP), normalized mean PI width (NMPIW), and coverage width-based criterion (CWC) are three indices for evaluating the correctness of the approximated PIs. The PICP reflects the degree of reliability of PIs and is defined as

(18) $$\mathrm{PICP}=\frac{1}{N}\sum_{t=1}^{N}I_{t}^{1-\alpha }$$

where $I_{t}^{1-\alpha }$ is defined as follows:

(19) $$I_{t}^{1-\alpha }=\{\begin{array}{c} 1\;u_{t}\in [L_{t}^{1-\alpha },U_{t}^{{}_{1-\alpha }}]\\ 0\;u_{t}\notin [L_{t}^{1-\alpha },U_{t}^{{}_{1-\alpha }}] \end{array}$$

NMPIW measures the width of the PI; it is defined as

(20) $$NMPIW=\frac{1}{N\varsigma }\sum_{t=1}^{N}\left(U_{t}^{1-\alpha }-L_{t}^{1-\alpha }\right)$$

where ς is the range of the underlying targets.

For high-quality PIs, narrow PIs (smaller NMPIW) with a high coverage probability (large PICP close to 100%) have great value [40,41]. Theoretically, NMPIW and PICP are conflicting. Therefore, CWC, which is a new balance criterion between PICP and NMPIW [42], is proposed to give a comprehensive assessment of PIs. CWC is defined as

(21) $$CWC=(NMPIW+\psi )e^{\frac{\gamma (PICP-\mu )}{2\delta^{2}}}$$

where ψ is a small positive value within the range of (0.1%, 0.5%), μ corresponds to the nominal confidence level associated with PIs that is usually set to 1-α, and δ is a small positive value less than 1. γ is set to 1 during the training process; for testing, it is defined by the following step function:

(22) $$\gamma =\{\begin{array}{c} 1,\;PICP\geq \mu \\ 0,\;PICP<\mu \end{array}$$

## 3. Case Study: Fanjiaping Landslide

### 3.1. Features of the Fanjiaping Landslide

**Figure 3:**
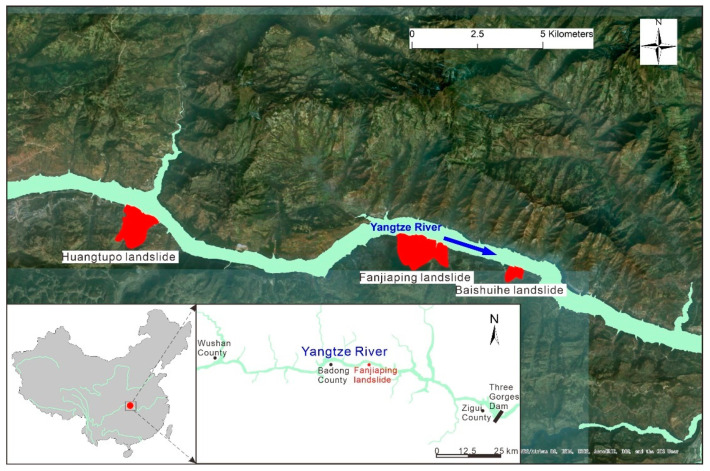
Location of the landslide site.

The Fanjiaping landslide is located on the southern bank of the Yangtze River and upstream of the Baishuihe landslide and downstream of the well-known Huangtupo landslide, which is approximately 56 km northwest of the Three Gorges Reservoir Dam (see Figure 3 for location). The Fanjiaping landslide is an ancient landslide [43,44] composed of two blocks: the Muyubao landslide and Fanjiaping landslide. The entire planar area of the landslide is approximately 1.96 million square meters, and the landslide volume is approximately 106 million cubic meters. The thickness of the Fanjiaping landslide ranges from 40 to 139.16 m. The Muyubao landslide is approximately 1500 m long and 1200 m wide. The average thickness of the Muyubao landslide body is approximately 50 m, and its estimated volume is 90 million m^3^.

**Figure 4:**
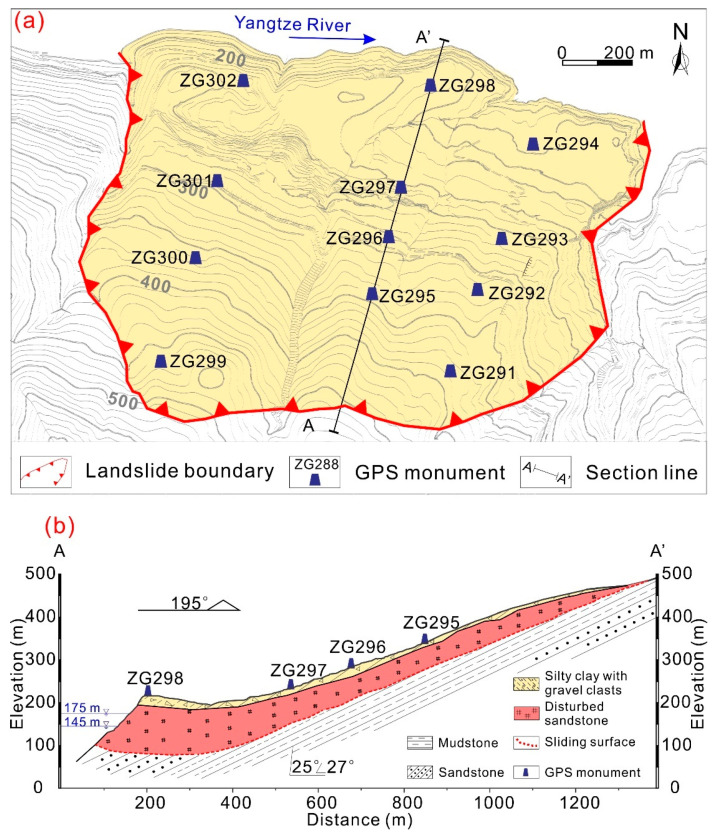

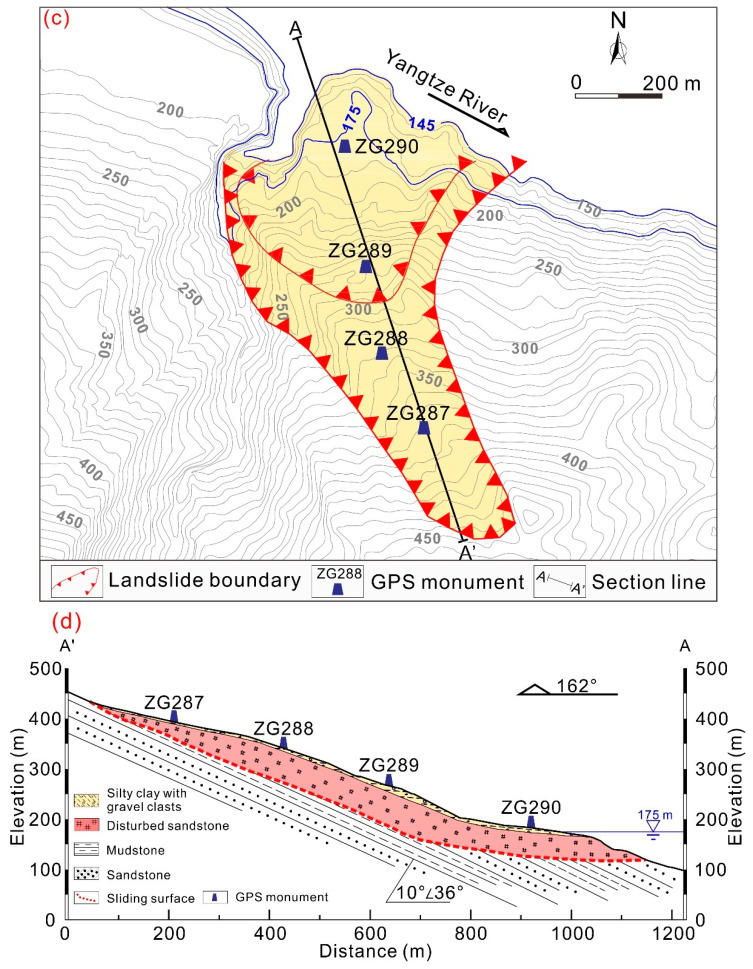
Topographic map and geological profile of the Fanjiaping landslide. (**a**) Topographic map of the Muyubao landslide. (**b**) Geological profile of the Muyubao landslide along sections A-A′, as recorded with monitoring instruments. (**c**) Topographic map of the Tanjiahe landslide. (**d**) Geological profile of the Tanjiahe landslide along sections B-B′, as recorded with monitoring instruments.

The Muyubao landslide extends from an elevation of 100 m at the toe to 520 m at the crown (Figure 4a,b). The slope surface consists of alternating gentle and comparatively steep landforms. The sliding direction of the landslide is 20°. The Tanjiahe landslide, located on the downstream of the Muyubao landslide, is approximately 1000 m long and 400 m wide. The average thickness of the Tanjiahe landslide body is approximately 40 m, and its estimated volume is 16 million m^3^. The Tanjiahe landslide extends from an elevation of 135 m at the toe to 420 m at the crown (Figure 4c,d). The slope surface consists of alternating gentle and comparatively steep landforms. The sliding direction of the landslide is 345°.

The site-specific investigation shows that the landslide materials are arranged in two different layers: a colluvial deposit at the upper surface and highly disturbed sandstone at the lower surface. The cataclastic sandstone is underlaid by sandstone and mudstone of the Jurassic Xiangxi formation (J1x) with an average dip direction of 10–25° and a dip angle of 27–36° (Figure 4b,d). Soft coal layers are prevalent in the J1x formation, and many landslides have developed along the soft coal layers. The borehole data indicates that the landslide mass of the Muyubao and Tanjiahe landslide slide along a soft coal layer with a thickness ranging from 0.1 to 0.3 m. According to laboratory testing of sliding zone soil obtained from the borehole, the natural moisture content of the soil is 12.6%, and the natural density is 1.9 g/cm^3^.

### 3.2. Input Data

**Figure 5:**
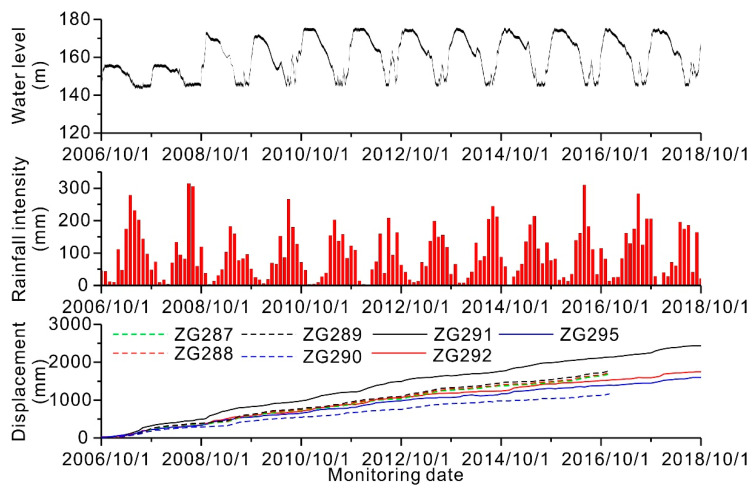
Reservoir water level, monthly rainfall intensity, and cumulative displacement from the Fanjiaping landslide area.

A total of sixteen GPS beacons were installed on the landslide mass to monitor the landslide movements in September 2006 (see Figure 4 for the GPS locations): four on the Tanjiahe landslide and twelve on the Muyubao landslide. The GPS monuments were manually surveyed once a month. In April 2016, four GPS monitoring points, ZG295, ZG296, ZG297, and ZG298, were updated to near real-time monitoring. At most, thirteen years’ worth of monitoring data were obtained. Figure 5 shows the monthly rainfall intensity obtained from the Shazhenxi Meteorological Station near the Fanjiaping landslide, the reservoir water level, and the displacement from GPS survey monuments over the thirteen-year period from October 2006 to March 2018. The available data indicate that the landslide was unstable and continuously deforming during the entire monitoring period. The landslide exhibits a step-like deformation behavior because of the periodic fluctuations in the reservoir water level and heavy precipitation. The monitoring data from both Muyubao and Tanjiahe show that larger displacements occurred in the upper middle part of the landslide mass. From the sequence of the surface cracks and displacement magnitude, we speculate that the movement occurred first at the rear part and progressed downslope. Based on a previous study on the relations between slip-surface geometry, material structures, and deformational structures [45,46], the observed kinematic behaviors are expected independent of the characteristics of the landslide material. However, more work is needed to confirm these findings.

### 3.3. Triggering Factors of the Landslide Movements

**Figure 6:**
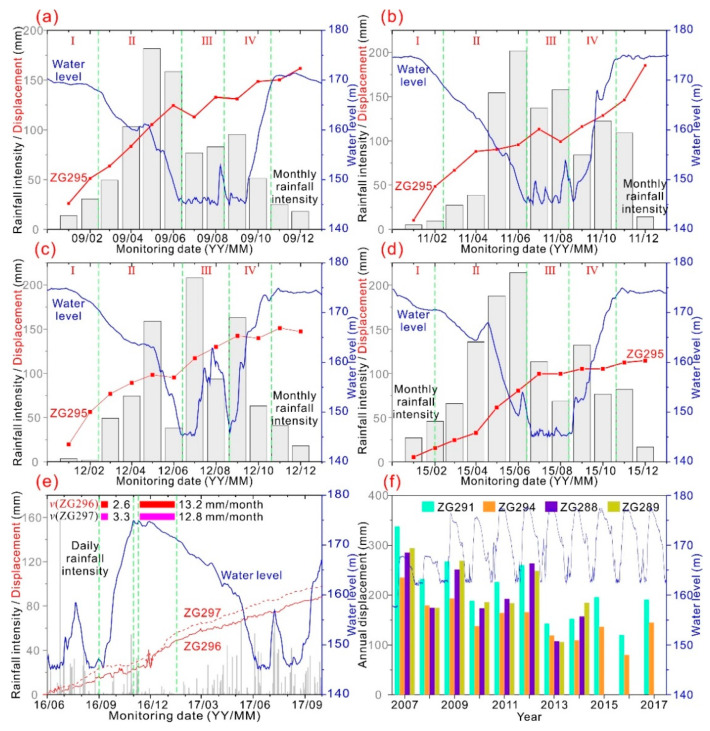
(**a**–**d**) Cumulative displacement at monitoring point ZG295, monthly rainfall intensity, and reservoir water level spanning the period of 2009, 2011, 2012, and 2015. (**e**) Cumulative displacement at monitoring points ZG296 and ZG297, daily rainfall intensity, and reservoir water level spanning the period of June 2016 to October 2017. (**f**) Annual displacement at monitoring point ZG291, ZG294, ZG288, and ZG289, and reservoir water level spanning the period of 2007 to 2017.

Although the Fanjiaping landslide is one of the largest landslides in the TGRA, very few publications have reported detailed information on the triggering factors of the landslide movements. Fully understanding the triggering factors is critical for landslide mitigation and early warning. In this study, long-term and near real-time monitoring data were used to comprehensively analyze the landslide movements. The cumulative displacement at monitoring point ZG295, monthly rainfall intensity, and reservoir water levels in 2009, 2011, 2012, 2015 are shown in Figure 6a–d. The available data shows the following trends:

(1) When the reservoir water level first rose from 135 to 156 m at the end of 2006, a significant annual displacement of 330 mm occurred at monitoring point ZG291 in 2007. Similarly, an annual displacement of 260 mm occurred at monitoring point ZG291 in 2009 when the reservoir water level rose from 156 to 172 m at the end of 2008. After 2009, the annual displacements shows a decreasing trend (Figure 6f). The results of the above analysis suggest that landslide deformation occurred at the preliminary operation phase, and more significant movement is likely to occur when the reservoir water level reaches a new higher level.

(2) A large deformation occurred when the reservoir water level slightly dropped from 175 m to 170 m in November to February (I in Figure 6). During 2009 to 2015, the monthly deformation rate during this drawdown period was greater than 20 mm per month (Figure 6a–d). For example, when the reservoir water level dropped from 174 m to 172.21 m in January 2012 to February 2012, the displacements measured at monitoring points ZG295, ZG296, ZG297, and ZG298 were 38.98, 26.39, 35.12, and 41.78 mm, respectively (Figure 6c). However, when the reservoir water level significantly dropped from 170 m to 145 m in February to June (II in Figure 6), the monthly deformation rate decreased to less than 20 mm per month.

(3) When the reservoir level remained at 145 m in July to September (III in Figure 6) and the landslide area suffered a heavy rainfall event, the landslide deformation was likely to be suspended except for 2012. The maximum monthly rainfall intensity during those suspended activities was 158 mm. In July 2012, the landslide area suffered from a heavy rainfall event with a monthly rainfall intensity of 208 mm, and the monitoring point deformed at a high rate. From those comparative analyses, we can speculate than the minimum triggering threshold consists of episodes lasting one month with cumulative rainfall exceeding 158 mm.

(4) When the reservoir rose from 145 m to 175 m in September to November (IV in Figure 6), monthly deformation rate decreased to a small positive (less than 10 mm per month) or even negative value.

(5) The near real-time monitoring data also showed the abovementioned trends: when the reservoir water level rose from 147.25 to 174.42 m on August 31 2016 to October 27 2016, the monthly deformation rates for ZG296 and ZG297 were 2.6 and 3.3 mm/month, respectively. When the reservoir dropped from 174.45 to 171.18 m on November 9 2016 to January 16 2017, deformations of 24.07 and 26.46 mm occurred at ZG296 and ZG297, respectively. The corresponding monthly deformation rates were 12.8 and 13.2 mm/month, respectively.

From the above analysis we can conclude that landslide movement was especially pronounced under prolonged periods of dropping reservoir levels, especially during periods of slight dropdown at the highest reservoir level, and the minimum triggering threshold consisted of episodes lasting one month, with cumulative rainfall exceeding 158 mm.

### 3.4. QRNNs-KDE-Based Method for Ensemble Prediction

#### 3.4.1. Data Splitting and Normalization

The available data (Figure 5) indicate that for the two active blocks, the largest displacements were observed at monitoring points ZG289 and ZG291, respectively. Therefore, monitoring points ZG289 and ZG291 were selected to establish a prediction model for the Fanjiaping landslide.

Previous correlation analysis in [3] revealed that weak to very strong correlations exist between landslide displacement and triggering and state variables. Therefore, based on triggering factor analysis and previous work on correlation analysis in [3], seven variables including four trigger variables and three state variables were selected as the inputs: rainfall intensity over the past month ($x_{1}(t)$), rainfall intensity over the past two months ($x_{2}(t)$), average reservoir water level in the current month ($x_{3}(t)$), variation in the reservoir water level in the current month ($x_{4}(t)$), displacement over the past one month ($x_{5}(t)$), displacement over the past two months ($x_{6}(t)$), and displacement over the past three months ($x_{7}(t)$). In addition, the displacement in the current month (Y(t)) was selected as the output. A data set $(x_{i}(t),Y(t)),i=1,2,\cdots ,7$ was generated based on the inputs and corresponding outputs. For the Tanjiahe landslide, the data from October 2006 to January 2015 with a size of 100 were treated as the training set, and the data from February 2015 to June 2015 with a size of 5 were used as the testing set. For the Muyubao landslide, the data from October 2006 to January 2015 with a size of 133 were treated as the training set, and the data from November 2017 to October 2018 with a size of 12 were used as the testing set.

#### 3.4.2. QRNN Modelling

Two nonlinear models with a sigmoidal transfer function and linear transfer function for τ = 0.01, 0.02, …, 0.98, 0.99 with an interval of 0.01 were trained for monitoring points ZG289 and ZG291 to generate multiple base learners. The number of hidden nodes in the QRNNs model was set to 5. The penalty for weight delay regularization was set to 1 to prevent overfitting in QRNNs model construction. For each monitoring period, a total of 99 base learners were obtained at conditional quantities ranging from 0.01 to 0.99 based on the QRNNs. The main parameters applied in the modelling of QRNNs are shown in Table 1.

#### 3.4.3. PDF Estimation by KDE

The multiple base learners from QRNNs were employed as inputs of Epanechnikov KDE to estimate the PDF. The optimal bandwidths for PDF estimation were calculated based on Silverman’s rule of thumb. The optimal bandwidths for PDF estimation of testing data at ZG289 were set to 7.98, 8.05, 5.66, 7.05, and 9.26. The optimal bandwidths for PDF estimation of testing data at ZG291 were set to 5.28, 8.61, 7.77, 5.62, and 5.30.

#### 3.4.4. Final Ensemble Prediction

Final ensemble predictions for the Fanjiaping landslide were generated through a probability combination scheme. PIs were constructed from the obtained PDF to estimate the predictive uncertainty. For the purpose of aiding decision-making, it is preferable to have prediction information with high confidence levels to reduce risks. Therefore, PIs at a high PINC value of 90% were obtained and analyzed in the study.

## 4. Results

**Figure 7:**
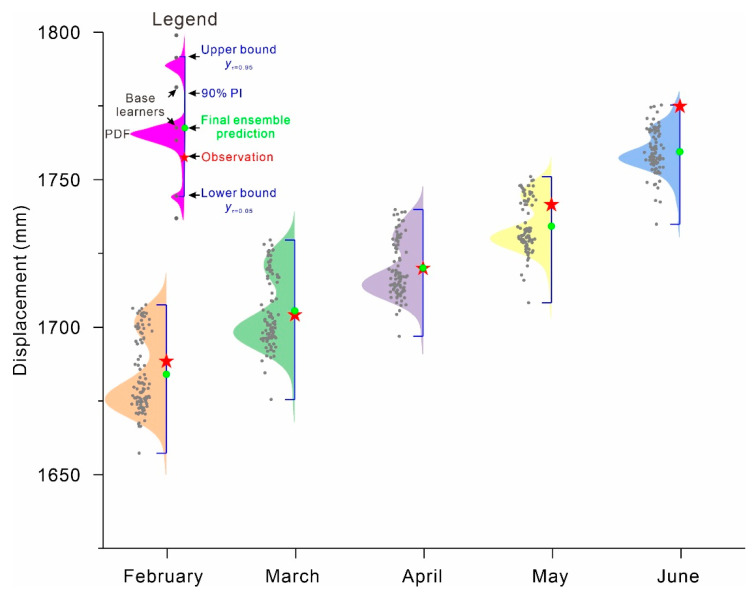
Probability density functions (PDFs) for the Fanjiaping landslide at ZG289 from February 2015 to June 2015.

**Figure 8:**
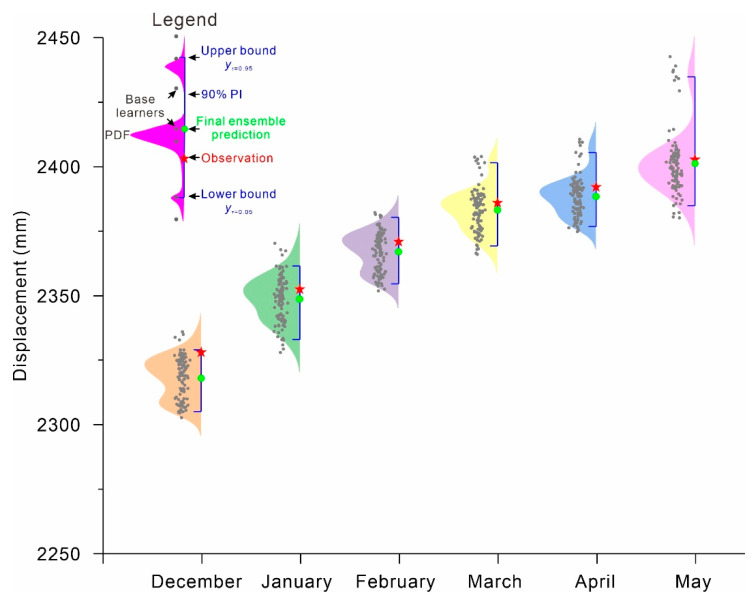
PDFs for the Fanjiaping landslide at ZG291 from December 2017 to May 2018.

PDFs: The PDFs of predictive displacement at ZG289 and ZG291 constructed by the proposed QRNNs-KDE approach are shown in Figure 7 and Figure 8. The fast movement is the main concern in landslide displacement prediction. Here, only a portion of the prediction describing the fast landslide is selected and shown. Figure 7 and Figure 8 show that rather than a single estimate, the range and complete PDF of the predictive displacement are provided by the proposed approach. All landslide displacement observations are distributed in the middle of the PDFs with high probability in addition to the observations of May and June at ZG289, which appear at the tail of the probability density curve. The small fraction falling into the right tail follows the increase in the prediction period; here, there are more uncertainties associated with longer-term landslide predictions.

**Figure 9:**
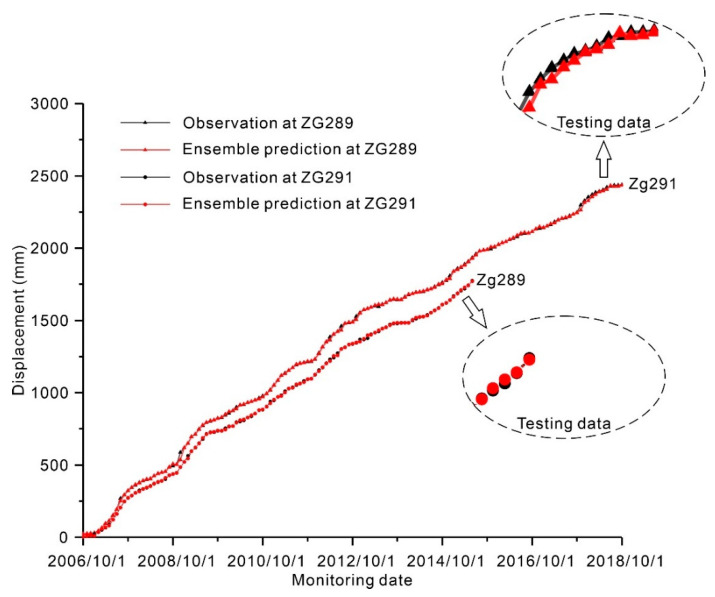
Comparisons of the final ensemble predictions and observations for the Fanjiaping landslide at ZG289 and ZG291.

Final ensemble prediction: Figure 9 shows the final ensemble predictions. As shown in Figure 9, the ensemble predictions obtained via the probability combination scheme showed a high degree of consistency in the landslide displacement observations, with coefficient of determination values of 0.999932 and 0.999944. To further evaluate the prediction performances of ensemble prediction based on the QRNNs-KDE, the evaluation metrics of the BP, RBF, ELM, and SVM approaches are shown in Table 2. As shown in Table 2, the final ensemble predictions using the QRNNs-KDE approach outperformed the persistence methods with the smallest MSE, RMSE, NRMSE, and MAPE and the largest R^2^. Moreover, compared with predictions at monitoring point ZG289 using the Copula-KSVMQR approach in [3], the QRNNs-KDE approach provided more accurate prediction with smaller MAPE and RMSE.

**Figure 10:**
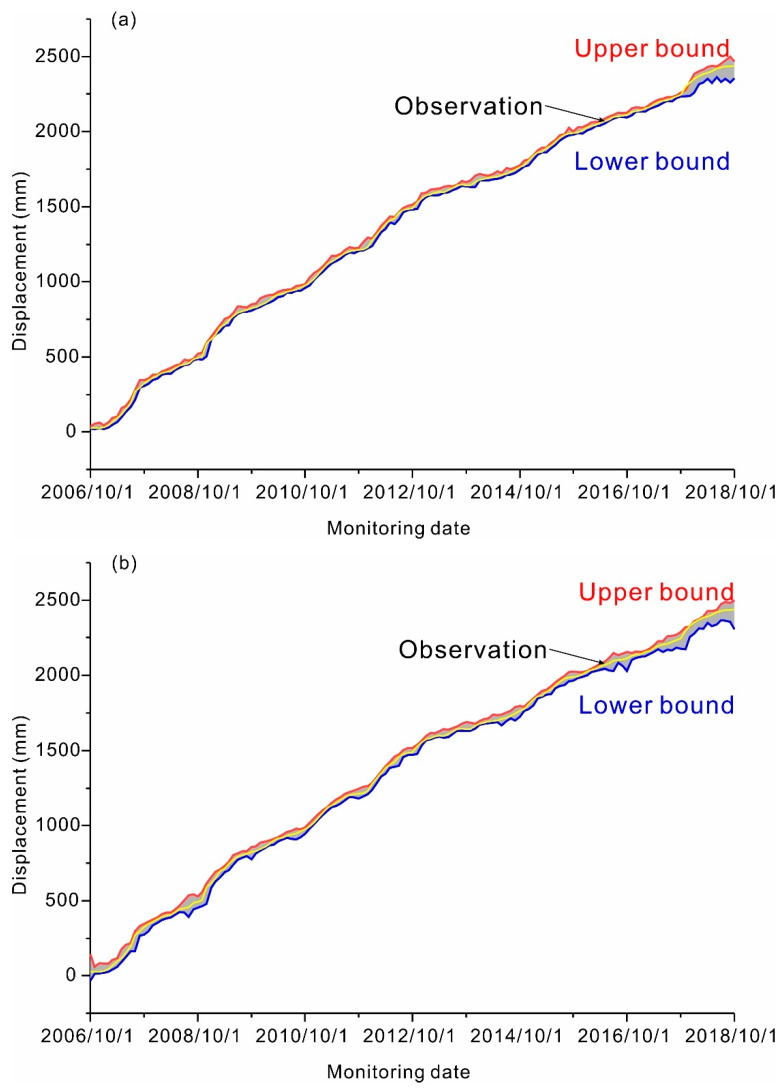

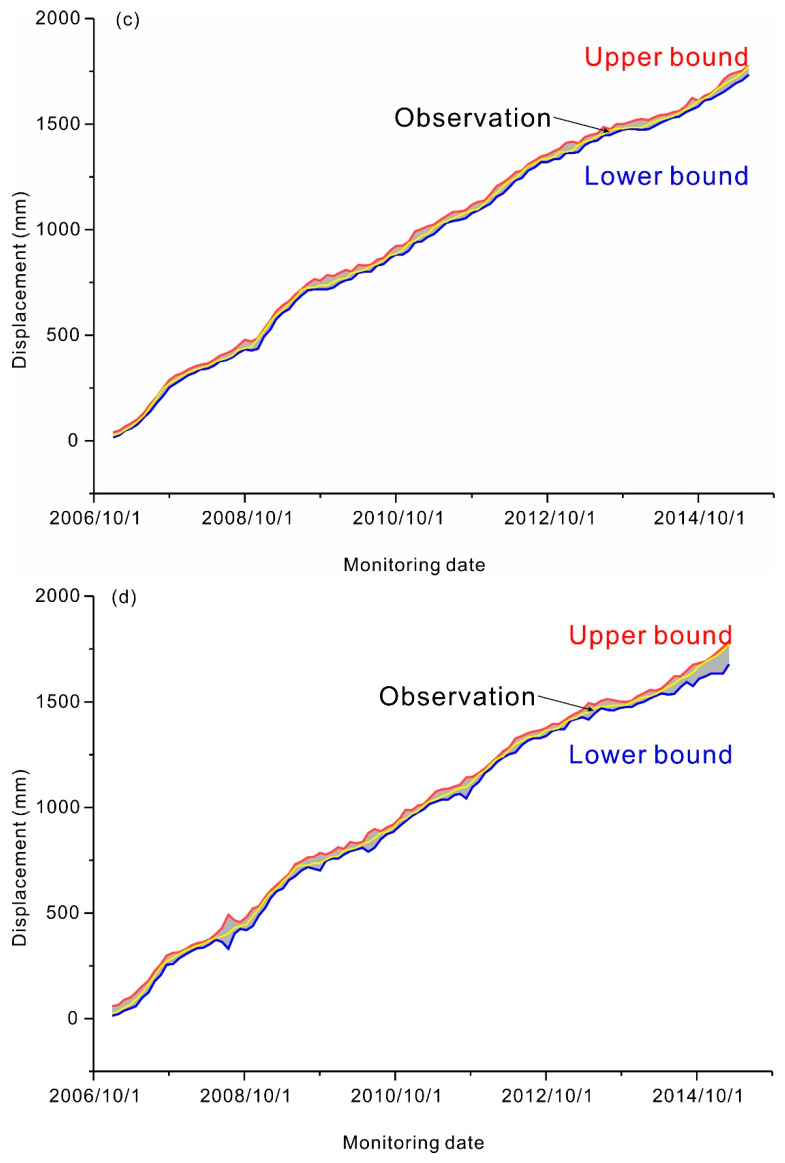
Comparisons of the observations and the constructed PIs at a 90% confidence level for the Fanjiaping landslide at ZG289 and ZG291 using QRNNs-KDE and bootstrap-ELM-ANN. (**a**) 90% PIs at ZG291 using QRNNs-KDE; (**b**) 90% PIs at ZG291 using bootstrap-ELM-ANN; (**c**) 90% PIs at ZG289 using QRNNs-KDE, (**d**) 90% PIs at ZG289 using bootstrap-ELM-ANN.

Uncertainty quantification: Based on the PDFs shown in Figure 7 and Figure 8, PIs at a high confidence level (90%) were constructed for ZG289 and ZG291 (Figure 10a,c, respectively). To evaluate the prediction performances based on the QRNNs-KDE approach, 90% PIs were constructed based on the bootstrap-ELM-ANN approach (Figure 10b,d). The corresponding evaluation metrics are shown in Table 3. As shown in Figure 10 and Table 3, the constructed PIs based on the QRNNs-KDE approach perfectly covered the observations with a high percentage, and the QRNNs-KDE approach outperformed the bootstrap-ELM-ANN approach with smaller NMPIW and CWC. For example, the performance indices NPIW and CWC of 90% PIs at ZG289 were 0.0215 and 0.1661, respectively, which were lower than those obtained using the bootstrap-ELM-ANN approach. The normalized mean PI width using the QRNNs-KDE approach was approximately 90% narrower than that for the bootstrap-ELM-ANN approach.

The experimental results show that the final ensemble predictions based on the QRNNs-KDE approach outperformed the traditional BP, RBF, ELM, SVM, and Copula-KSVMQR algorithms with regard to deterministic point prediction. The QRNNs-KDE approach was more informative than traditional algorithms because it provided the likely range of landslide displacement. The landslide observations were distributed in the middle of the prediction range with high probability. Moreover, regarding the aspect of uncertainty quantification, the QRNNs-KDE provided more satisfactory PIs than the bootstrap-ELM-ANN approach. Therefore, we believe that the final ensemble predictions based on the QRNNs-KDE approach have the advantages of accurate prediction and uncertainty quantification of landslide displacement.

## 5. Discussion

**Figure 11:**
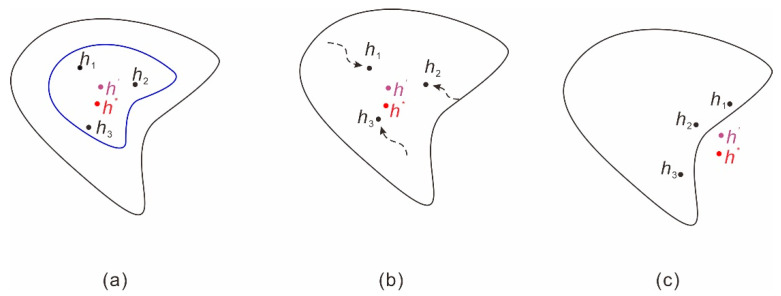
Schematic that shows the fundamental benefits of the ensemble prediction model from statistical (**a**), computational (**b**), and representational (**c**) perspectives. h^*^ is the true prediction model; *h*_1_, *h*_2_, and *h*_3_ are single prediction models; and *h*^’^ is the ensemble prediction model obtained by combining the single prediction models *h*_1_, *h*_2_, and *h*_3_. The outer black curve is the hypothesis space of all possible models. The inner blue curve denotes the subset of hypotheses that give reasonable accuracy with the available training data (modified from [47]).

In this study, with regard to point prediction, the probability-scheme combination ensemble prediction, which employs QRNNs-KDE, provided the best prediction. The fundamental reasons behind this can be explained from statistical, computational, and representational perspectives [47]. From a statistical perspective, the available training data set may not be able to provide sufficient information for training the true model (h^*^ in Figure 11). Constructing an ensemble model (*h*^’^ in Figure 11) might not be better than the single best prediction model h^*^, but it does reduce the risk of choosing a bad learner with poor generalizability (schematic in Figure 11a). From a computational perspective, in a single model the training algorithms might get stuck in lock optima by only performing a local search. Constructing an ensemble model by searching from different starting positions might be a better alternative (schematic in Figure 11b). From a representational perspective, it is possible that the searched hypothesis space might not contain the true model h^*^. Constructing an ensemble model might expand the representable space (schematic in Figure 11c).

In the proposed QRNNs-KDE approach, the probability combination scheme is employed to combine 99 base learners into one final ensemble to improve the model performance. However, a concern about computational time may be associated with this ensemble strategy. The required computational time is highly related to the number of base learners. For the case of ZG 291, the required computation time is 191.85 s to train 99 base learners in RStudio Version 1.2.5042 on an Intel(R) Xeon(R) E-2176M @ 2.70 GHz CPU with 64 GB RAM. Thus, we believe that the proposed approach is computationally efficient.

Nevertheless, the probability-scheme combination ensemble prediction, which employ QRNNs and KDE, also holds inherent limitations associated with data-driven models, such as the lack of an explicit input-output relationship, and the requirement of large training data to maintain the model performance.

In practical applications, the main motivation for the construction of predictive range and complete PDF is to quantify the likely predictive uncertainty in the deterministic point predictions. Availability of range and complete PDF of the predictive displacement allows the researchers and practitioners to efficiently quantify the level of predictive uncertainty with the deterministic point predictions and to consider a multiple of solutions/scenarios for the best and worst conditions. Wide ranges are an indication of presence of a high level of uncertainty in the operation. This information can guide the researchers and practitioners to avoid the selection of risky actions under uncertain conditions. In contrast, narrow range means that decisions can be made more confidently with less chance of confronting an unexpected condition in the future, for example, if a sharp displacement increment with a wider range was predicted for the further. An alert should be carefully determined whether reaching tertiary creep stage by researchers and practitioners through comprehensive analysis. Under this circumstance, time-of-failure forecasting should be run in parallel, and a multiple of solutions/scenarios should be considered until either failure precursors are identified or the movements suspended.

The proposed QRNNs-KDE approach is suitable for medium-term to long-term horizon forecasting. Results from previous studies [2,48] have shown that the performance of data-driven models varies for landslides with different deformation behaviors. Usually, for landslides with drastic step-like deformation, the prediction accuracy is lower, and the corresponding prediction error is larger. Therefore, in practical applications of medium-term to long-term horizon forecasting, when predicting landslides with drastic deformation, the proposed QRNNs-KDE approach should be applied with caution. To achieve excellent performance, sufficient data are recommended and needed for model training.

## 6. Conclusions

In this study, a QRNNs-KDE approach was proposed to improve the prediction accuracy and uncertainty quantification of landslide displacement. The Fanjiaping landslide in the TGRA was selected as a case study to explore the performance of the QRNNs-KDE approach. The following conclusions from the study were obtained:

The movements of the Fanjiaping landslide was especially pronounced under prolonged periods of dropping reservoir levels, especially during periods of slight dropdown at the highest reservoir level, and the minimum triggering threshold consists of episodes lasting one month, with cumulative rainfall exceeding 158 mm.

The QRNNs-KDE approach achieves perfect performance and outperforms the traditional BP, RBF, ELM, SVM, bootstrap-ELM-ANN, and Copula-KSVMQR methods. Additionally, the proposed approach is more informative by providing the likely range and complete PDFs of landslide displacement. The landslide displacement observations are distributed in the middle of the prediction range with high probability.

In practical application, the proposed QRNNs-KDE approach is suitable for medium-term to long-term horizon forecasting. The range and complete PDF of the predictive displacement can supplement final point predictions for decision making.

## Figures and Tables

**Table 1:** The parameters utilized in the QRNNs modeling for the Fanjiaping landslide.

Parameter	Value	Parameter	Value
Maximum number of iterations	5000	Penalty for weight decay regularization	1
Number of quantiles	99	Number of input nodes	7
Number of repeated trials	5	Number of hidden nodes	5

**Table 2:** Comparisons of predictions obtained from QRNNs-KDE, BP, RBF, ELM, and SVM for the Fanjiaping landslide.

Monitoring Point		Model	BP	RBF	ELM	SVM	QRNNs-KDE
	Index						
ZG289	R^2^		0.99730	0.99992	0.99785	0.99993	***0.99997***
	MSE		3192.07	99.54	2538.74	78.12	***30.69***
	RMSE		56.50	9.98	50.39	8.84	***5.54***
	NRMSE		0.032263	0.005697	0.028772	0.005047	***0.003163***
	MAPE		2.74	2.00	1.57	1.27	***1.17***
ZG291	R^2^		0.99991	0.99759	0.99991	0.99995	***0.99997***
	MSE		206.32	5684.98	215.41	119.75	***70.15***
	RMSE		14.36	75.40	14.68	10.94	***8.38***
	NRMSE		0.005953	0.031251	0.006083	0.004536	***0.003471***
	MAPE		3.97	1.96	2.59	2.33	***0.41***

*Note*: The most accurate prediction results are shown in bold italics.

**Table 3:** Comparisons of 90% PIs obtained from Bootstrap-ELM-ANN and QRNNs-KDE for the Fanjiaping landslide.

Monitoring Point		Index	PICP	NPIW	CWC
	Model				
ZG289	Bootstrap-ELM-ANN		100%	0.27	0.2071
	***QRNNs-KDE***		***100%***	***0.0215***	***0.1661***
ZG291	Bootstrap-ELM-ANN		99%	0.024	0.143
	***QRNNs-KDE***		***99%***	***0.018***	***0.085***

*Note*: The prediction results with a narrower PI range are shown in bold italics.
